# A Pilot Study on Biochemical Profile of Follicular Fluid in Breast Cancer Patients

**DOI:** 10.3390/metabo13030441

**Published:** 2023-03-17

**Authors:** Maria A. Castiglione Morelli, Assunta Iuliano, Ilenia Matera, Licia Viggiani, Sergio C. A. Schettini, Paola Colucci, Angela Ostuni

**Affiliations:** 1 Department of Sciences, University of Basilicata, 85100 Potenza, Italy; 2 Center for Reproductive Medicine of “San Carlo” Hospital, 85100 Potenza, Italy

**Keywords:** NMR-based metabolomics, breast cancer, fertility preservation, oxidative stress, inflammation, tumor stage

## Abstract

Breast cancer (BC) is the most common type of cancer among women in almost all countries worldwide and is one of the oncological pathologies for which is indicated fertility preservation, a type of procedure used to help keep a person’s ability to have children. Follicular fluid (FF) is a major component of oocyte microenvironment, which is involved in oocyte growth, follicular maturation, and in communication between germ and somatic cells; furthermore, it accumulates all metabolites during oocytes growth. To obtain information about changes on fertility due to cancer, we aimed at investigating potential biomarkers to discriminate between FF samples obtained from 16 BC patients and 10 healthy women undergoing in vitro fertilization treatments. An NMR-based metabolomics approach was performed to investigate the FF metabolic profiles; ELISA and western blotting assays were used to investigate protein markers of oxidative and inflammatory stress, which are processes closely related to cancer. Our results seem to suggest that FFs of BC women display some significant metabolic alterations in comparison to healthy controls, and these variations are also related with tumor staging.

## 1. Introduction

Breast cancer (BC) is the most common type of cancer among women in almost all countries worldwide and affects approximately 10% of all women at some stages of their life [1]. According to GLOBOCAN (2020), BC is the most commonly diagnosed cancer, with an estimated 2.3 million new cases in 2020 [2].

BC is a heterogeneous disease originating from the epithelial cells lining the milk ducts. Its heterogeneity was noted in histology and clinical outcome, and these differences were used as the basis for disease classification [3]. The traditional pathology-driven classification has been refined and replaced by molecular classifications, so to combine disease mechanisms with the design of individualized therapies, leading to significant improvements in disease-specific survival [4]. In clinical practice BC is usually classified into three groups: hormonal estrogen (ER+) and progesterone receptor-positive (PR+), human epidermal growth factor receptor positive (HER2+) and triple negative (TNBC), that lacks expression of all these three receptors and is considered the most fatal form of BC.

BC may be described as a multi-step process, where each step is correlated with mutations in major regulatory genes [5]. BC may be sporadic or hereditary, where sporadic BC results from a serial stepwise accumulation of acquired and uncorrected mutations in somatic genes, with no germline mutations. Hereditary BC is characterized by an inherited susceptibility to breast cancer on the basis of an identified germline mutation in BRCA1 and BRCA2 tumor suppressor genes, involved in double strand DNA break repairs, or in one allele of high penetrance susceptibility genes (as TP53, CDH1, PTEN, …) or low/moderate-penetrance genes (such as *ATM*, *CHEK2* and others) [6]. 

BC is one of the oncological pathologies for which is indicated fertility preservation [7], a type of procedure often applied in oncology to give an opportunity to cancer survivors to maintain reproductive health and have children after oncologic treatment [8,9,10]. Chemotherapy, radiotherapy, surgery or a combination thereof expose women of reproductive age to risk of damage to the ovary, increase the risks of premature ovarian failure (POF), early menopause, ovarian endocrine disorders, and infertility [11,12,13].

No alterations in the quantity and quality of oocytes were found in some cohorts of patients affected by BC [14,15]. Quinn et al. [16] demonstrated that BC patients do not have compromised ovarian reserve nor do they have a different response to ovarian stimulation or oocyte yield when compared to healthy women. Moreover, on their cohort of patients, cancer stage did not have any impact on ovarian stimulation outcomes. 

The metabolic characteristics of cancer cells change during disease progression, and this is reflected in their metabolic profiles. Metabolomics is applied in oncology to study cancer metabolism and for characterizing metabolic profiles associated with oncogenotypes, disease progression, and therapeutic targets [17]. 

Mass spectrometry and Nuclear Magnetic Resonance spectroscopy (NMR) are the two most widely used analytical platforms in metabolomics studies and can be considered as two complementary techniques to get information about the whole set of metabolites in a biological system [18]. Furthermore, Magnetic Resonance Imaging (MRI) represents a powerful technique widely used in the diagnostic and treatment evaluation of breast cancer [19,20].

Detecting cancer by metabolic profiling of biofluids facilitates easy and minimally invasive diagnostics, and allows for screening [21]. Metabolomics has been widely utilized in BC field [22,23,24,25,26,27,28,29] using different type of samples (i.e., blood plasma/serum, saliva, tissues, urine, and cells). 

Follicular fluid (FF) is derived from ovarian follicles, which contain many types of metabolites that are involved in many critical processes of oocyte maturation and development. Besides, FF is a superfluous product, easily available during oocyte pick-up in in vitro fertilization (IVF), and represents an optimal source of biochemical predictors of oocyte quality [30]. Few years ago, Piñero-Sagredo et al. studied the NMR metabolic profile of human FF from healthy oocyte donors and identified some of the metabolites present [31]. Other studies have utilized NMR metabolomic approaches to associate the FF metabolic profile with oocyte quality and implantation outcome [32] or with infertility diseases [33,34,35,36]. Previously, we demonstrated that FF has a different metabolic composition in cancer patients and healthy controls [37]. 

In order to obtain more information about any changes on fertility due to cancer itself, this study aims to identify potential biomarkers to discriminate between FF samples obtained from breast cancer patients and healthy women undergoing **IVF** treatments. An NMR-based approach was performed to investigate the FF metabolic profiles; ELISA and western blotting assays were used to investigate protein markers of oxidative and inflammatory stress, events closely related to cancer. 

## 2. Materials and Methods

### 2.1. Patients 

The local Ethics Committee (Comitato Etico Unico Regionale per la Basilicata) approved this study on October 2015, (approval number: 82/2015 of 7 October 2015). All participants gave written informed consent to the study, according to the Declaration of Helsinki.

26 women were enrolled in the study between 2019 and 2022. A first group consisted of 16 BC patients with estrogen-sensitive tumors. They underwent oocyte cryopreservation because cancer therapy may negatively impact their reproductive potential. The inclusion and exclusion criteria used for selecting the oncological participants are reported elsewhere [37]. For all BC patients we had tumor stadiation data; only for 9 of them we have also some molecular classification information. The other women were from outside Basilicata region and, after oocyte cryopreservation, they were followed for cancer treatments in hospitals located in their residing area. 

The second group consisted of 10 healthy women undergoing treatment for IVF at the Center for Reproductive Medicine. These women did not suffer from cancer or other diseases. Their infertility indication was by mild to moderate male factor. 

The clinical information of participants in the study is summarized in Table 1.

All patients performed ovarian reserve tests: basal FSH, AMH, and antral follicle count before starting ovarian stimulation to have homogeneous samples with respect to the ovarian reserve. Healthy controls received stimulation with recombinant follicle-stimulating hormone (FSH) (Gonal-f, Merck Serono or Ovaleap, Theramex) or urinary highly purified FSH (Fostimon, IBSA) and gonadotropin-releasing hormone (GnRH) antagonist (Cetrotide, Merk Serono or Fyremadel, Ferring). 

BC patients received a different ovarian hyperstimulation protocol: they followed the Oktay protocol [38] where aromatase inhibitors (Femara 2.5 mg, Novartis) are used to modulate the adverse effects of high estradiol level, in association with gonadotropins and gonadotropin-releasing hormone (GnRH) antagonist. On day 2 of the menstrual cycle, a FSH dose was used according to the nomogram of La Marca [39] for follicular stimulation in all women. The monitoring of follicular growth, and the triggering of the ovulation were performed as previously described [37]. The oocytes retrieval was performed after 34–36 h after triggering. Cumulus-oocyte complexes and FF were collected via transvaginal ultrasound-guided aspiration. 

### 2.2. Sample Preparation and NMR Analysis

Preparation of the samples and ^1^H NMR spectroscopy experiments were performed as previously reported [37]. All the spectra were processed with the software ACD/1D NMR Processor (version 12.01, Academic Edition, ACD Labs, Toronto, Ontario, Canada) and the integral buckets were used as input data for subsequent multivariate analyses [37].

The identification of metabolites responsible for sample differentiation was accomplished using data from literature [31] in combination with the data banks HMDB (http://www.hmdb.ca/, accessed on 10 October 2022) and BMRB (http://www.bmrb.wisc.edu/metabolomics/, accessed on 19 October 2022).

#### Multivariate Analysis of NMR Data

Multivariate data analyses were performed using SIMPCA-P+ software (version 12, Umetrics, Sweden). Principal Component Analysis (PCA) and Projection to Latent Structures regression Discriminant Analysis (PLS-DA) models were calculated. The results of cross validation for PLS-DA models were given by cumulative R^2^ and Q^2^ values. The PLS-DA models were validated using permutation tests.

The heatmaps were calculated with Morpheus software (https://software.broadinstitute.org/morpheus, accessed on 10 December 2022).

### 2.3. Western Blot Analysis

Five micrograms of proteins from follicular fluids were separated by 15% SDS-PAGE and electrotransfered to a nitrocellulose membrane (Amersham Bioscience, Buckinghamshire, UK). Membranes were blocked with 5% milk for 1 h at room temperature and incubated overnight at 4 °C using the following primary antibodies: 1:100 Anti-SOD2 (sc-130345) (Santa Cruz Biotechnology, Inc., Dallas, TX, USA), 1:100 Anti-Nrf2 (sc-365949) (Santa Cruz Biotechnology, Inc., Dallas, TX, USA), 1:100 Anti-NQO1 (sc-32793) (Santa Cruz Biotechnology, Inc., Dallas, TX, USA); subsequently were incubated with the appropriate horseradish peroxidase-conjugated secondary antibodies. The signals were visualized by ECL™ Western Blotting Detection Reagents (Amersham Bioscience, Buckinghamshire, United Kingdom). All blots were imaged on Chemidoc™ XRS detection system equipped with Image Lab Software for image acquisition (BioRad, Hercules, California, USA) and processed using GelAnalizer 19.1 software (www.gelanalyzer.com by Istvan Lazar). Specific immunoreactive bands were normalized to total protein by staining membranes with Ponceau S solution (Sigma, Saint Louis, MO, USA). All experiments were replicated three times. 

### 2.4. Measurement of Chemokine CXCL10 

To evaluate the concentration of CXCL10 in the follicular fluids, the ELISA kit CXCL10/IP-10 (Proteintech, Am Klopferspitz, Planegg-Martinsried, Germany) was used, according to the manufacturers’ instructions. 

### 2.5. Statistical Analysis

Normally distributed clinical data were compared across study groups by univariate ANOVA (Systat 11.0, Systat Software, Inc., San Jose, CA, USA). Pairwise comparisons of the means were performed with Fisher’s least significant differences (LSD) test. The minimum level of statistical significance was *p* < 0.05. Values are presented as mean ± standard deviation (SD).

Data of western blot and ELISA analysis were analyzed using GraphPad Prism 8.0 software and presented as mean ± standard error of three independent experiments. Statistical analysis was performed by using *t*-test; * *p* < 0.05, comparing follicular fluid from breast cancer patients vs. follicular fluid from healthy control. After normality testing of data, one-way ANOVA analysis was used for comparison among three groups: healthy controls and BC patients, subdivided in two sub-groups, without and with lymph node metastasis. Post-hoc Tukey test was performed to evaluate the significance of the observed differences.

### 2.6. Analysis of Discriminating Capability of Metabolites and Proteins

The performance of the identified metabolites and proteins as biomarkers to discriminate breast cancer patients and controls was further assessed using receiver operating characteristics (ROC) analysis. It was calculated with the Biomarker Analysis module of MetaboAnalyst 5.0 (https://www.metaboanalyst.ca/MetaboAnalyst/home.xhtml, accessed on 5 November 2022).

## 3. Results

### 3.1. Patient’s Characteristics

A total of 16 female breast cancer patients were enrolled in the study with a median age of 34 (23–43) years. As a control we selected 10 healthy women undergoing treatment for IVF with a median age of 36.5 (28–42) years. The clinical features of all the subjects are shown in Table 1. Only estradiol resulted significantly different (*p* < 0.05) in the two groups with lower levels observed in BC women thanks to the administration of aromatase inhibitors (letrozole) in women with hormone-sensitive breast cancer [13]. No other significant differences in clinical parameters were found.

The lymph node metastasis was observed in 6 (37.5%) patients, while 10 (62.5%) patients were found to be nodal negative. All the BC patients did not present distant metastasis. Overall, out of 16 breast cancer patients, 13 (81%) were in the early (I + II) pTNM stage and 3 (19%) were found to be in the late stage (III) of cancer (Table 1).

At the present time, the oocytes of all our BC patients are still cryostored, so we lack of subsequent embryo and pregnancy data.

### 3.2. Identification of Biomarkers in BC Patients 

**Figure 1:**
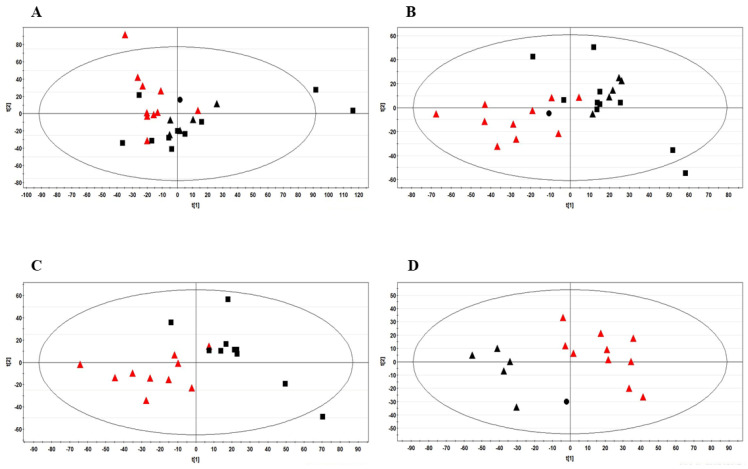
(**A**) PCA and (**B**) PLS-DA score plots obtained by ^1^H-NMR analysis on FF samples from all the 26 women examined in the study. The R^2^X and Q^2^ values for the two-component PLS-DA model were: 0.35 and 0.34, respectively. PLS-DA score plot between healthy women (n = 10) and: (**C**) BC patients with no lymph node metastasis (n = 10); (**D**) BC patients with lymph node metastasis (n = 6). The R^2^X and Q^2^ values for the two-component model reported in (**C**) were: 0.37 and 0.32, respectively; the corresponding values for the two-component model shown in (**D**) were 0.36 and 0.54, respectively. Data were colored by group: healthy controls were represented with red triangles; BC patients are shown in black using different symbols: patients with no lymph node metastasis (n = 10), boxes; patients with N1 (n = 5) and N2 lymph node metastasis (n = 1), triangles and dot, respectively.

One-dimensional ^1^H NMR spectra were acquired on FF samples from BC patients, together with healthy controls (Figure S1). To confirm the observed differences in the spectra of the two groups, multivariate analysis on the NMR data was carried out. The unsupervised principal component analysis (PCA) was initially done to gain a tendency of separation of samples according to groups (Figure 1A). For further separation of groups, PLS-DA models were generated: a first model compared all the BC patients with healthy women (Figure 1B); a second model was built by setting healthy controls against BC patients subdivided in two sub-groups, without (N0) and with lymph node metastasis (N1 + N2) (Figure S2). In addition, pair-wise comparisons were performed on the two lymph node metastasis sub-groups respect to healthy controls. The results are shown in Figure 1C,D, respectively.

**Figure 2:**
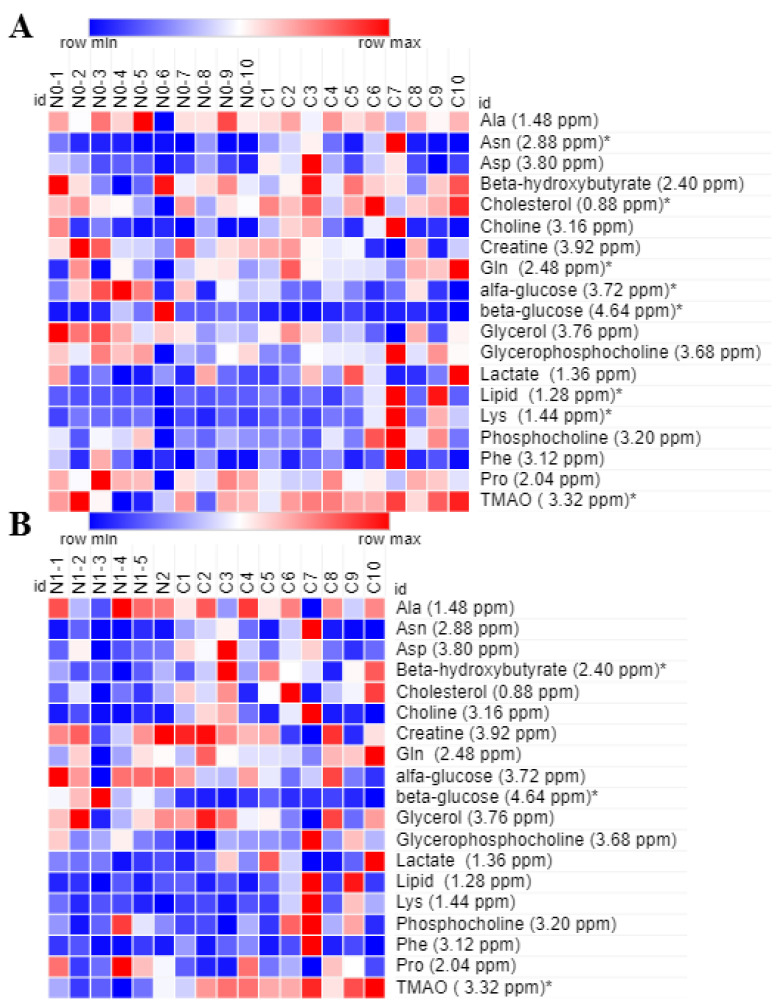
Heatmaps of the most relevant metabolites (with a VIP value > 1) that were associated with the differentiation between healthy controls (n = 10, group C) and: (**A**) BC patients with no lymph node metastasis (n = 10, group N0); and (**B**) BC patients with lymph node metastasis (n = 6, group N1 + N2). In columns are shown the different groups of women; in rows is reported the quantification of NMR integral bin regions of metabolites with VIP > 1, an * is used where significant differences between groups are observed (*p*-values < 0.05). Color scale indicates values ranging from blue (the lowest) to red (the highest).

Furthermore, PLS-DA generated a list of 16 signals with VIP (Variable Importance in the Projection) values > 1, which represent 13 potential metabolites useful for the discrimination of the groups (Supplementary Table S1). Seven metabolites resulted significantly different in the group of BC women with no lymph node metastasis in comparison to healthy controls: higher levels of glucose were found while the levels of some amino acids (Asn, Gln, Lys) were lower together with those of lipids, cholesterol and TMAO (Figure 2A). However, TMAO was not very important for discriminating the two groups (VIP < 1). Significant differences were also observed between the six BC women with lymph node metastasis and healthy controls: in the first group of women, the levels of β-hydroxybutyrate and TMAO were lower and levels of glucose were higher, as also observed in BC women with no lymph node metastasis (Figure 2B).

Although the number of subjects included in this study is quite limited, our results suggest that in comparison to healthy controls, the FFs of BC women display some metabolic alterations related with tumor staging.

### 3.3. Follicular Fluid Antioxidant and Anti-Inflammation Biomarkers

**Figure 3:**
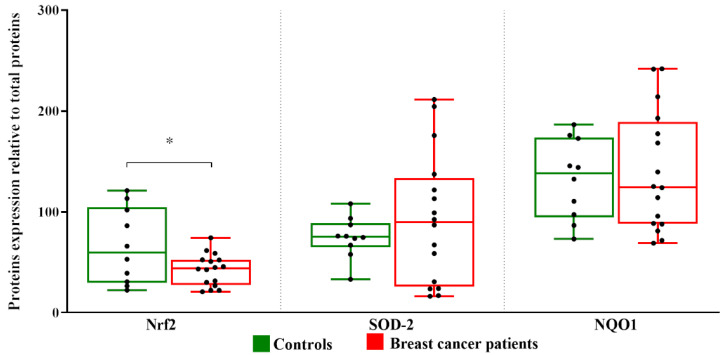
Expression level of Nrf2, SOD-2, and NQO1 proteins in follicular fluids. Densitometric analysis of the immunoreactive bands performed in three independent experiments. After densitometric analysis, western blot signals of the target proteins are normalized to the total amount of protein in each lane. The box plots show medians and whiskers. Significance (* *p* < 0.05).

Nuclear factor erythroid 2-related Factor 2 (Nrf2) is an ubiquitous master transcription factor that under stressful conditions upregulates the expression of antioxidant enzymes and cytoprotective proteins including NADPH quinone oxidoreductase1 (NQO-1) and superoxide dismutase (SOD) [40]. In FF of BC patients, a decrease in Nrf2 expression level is observed in comparison with healthy controls which supports no change observed neither in NQO1 nor in SOD-levels (Figure 3).

In addition, Nrf2 is also a key transcription factor in anti-inflammatory pathways through a functional cross-talk with NF-kB signaling thus leading to enhance or impair the inflammatory response, depending on the specific context and stimuli Cytokines and chemokines are involved in alterations of the cellular redox state and in inflammatory processes [41]. We evaluated a possible alteration of cytokines and chemokines in the FF of BC patients by ELISA assays. No significant changes are observed in the mean levels of the proinflammatory cytokines IL-6 and TNF-α in the FF of BC patients compared to those found in healthy women. 

**Figure 4:**
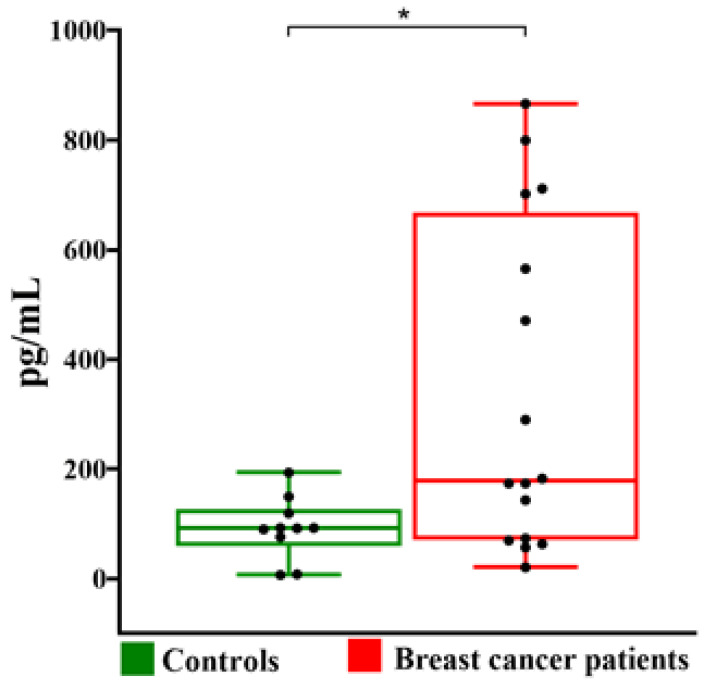
Boxplot representation of CXCL10 level in follicular fluids of control and BC patients. The box plots show medians and whiskers. Significance (* *p* < 0.05).

ELISA assay showed that the concentrations of chemokine CXCL10 in follicular fluid obtained from BC patients were significantly higher than those obtained from healthy control patients (Figure 4).

Then we analyzed the levels of both Nrf2 and CXCL10 measured in follicular fluids of the study participants divided in three groups: healthy controls, BC patients without lymph node metastasis and with lymph node metastasis. Only the levels of CXCL10 were significantly different in the three groups (*p* = 0.029). 

### 3.4. Assessment of Diagnostic Potential and Predictive Ability of Some Identified Biomarkers

**Figure 5:**
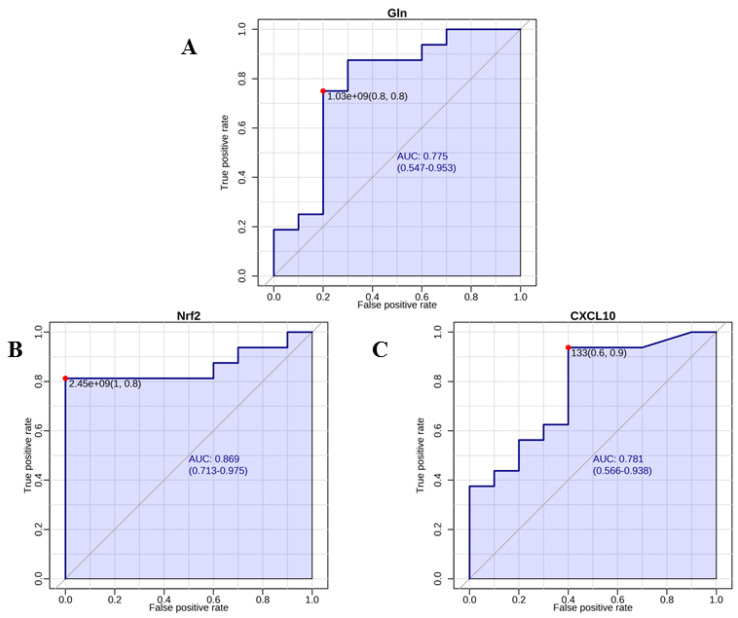
ROC curves for glutamine (**A**), and the 2 proteins or Nrf2 (**B**) and CXCL10 (**C**) with the highest ability to discriminate BC patients against controls. The curves were obtained with MetaboAnalyst 5.0 (https://www.metaboanalyst.ca/MetaboAnalyst/home.xhtml, accessed on 5 November 2022).

ROC curve analysis was performed to determine the diagnostic potential of NMR-identified metabolites and of the Nrf2 and CXL10 proteins (Figure 5). The area under curve (AUC) was used to assess the performance of predicted biomarkers. Among the metabolites with VIP values > 1, only Gln was found to have a fair predictive ability in discriminating FFs of BC patients from healthy controls, with AUC = 0.775 (*p* = 0.002) (Figure 5A). In addition, also the proteins Nrf2 and CXL10 had a fair predictive ability, with AUC = 0.869 (*p* = 0.0004) and AUC = 0.725 (*p* = 0.02), respectively (Figure 5B,C).

## 4. Discussion

Follicular fluid contains a variety of autocrine and paracrine factors responsible for the regulation of folliculogenesis, oocyte development, and ovarian function [42]. 

Study of FF allows the analysis of a wide range of metabolites from different pathways. The composition of FF undergoes physiological alterations during follicular development [43], in women undergoing controlled ovarian stimulation for IVF [44,45,46] as well as in different pathological conditions [47,48]. As far as we know, little has been investigated on changes in the FF composition due to the direct effect of the tumor itself or as a consequence of the tumor, including cancer [14,15,16,37]. In the context of the issue of fertility preservation offered to women affected by breast cancer before undergoing chemo/radiotherapy, it is a priority to evaluate the impact of systemic conditions on the follicular fluid composition and definitely on the quality of oocytes which are generally cryopreserved in awaiting fertilization. 

A different metabolic profile was observed in FF of our BC patients compared with the healthy controls. Among the altered metabolites, we found out higher level of glucose and lower levels of lactate and Gln. The levels of Gln were found significantly decreased also in other studies on patients with BC [49,50] and the decrease of Gln might imply a dysregulation in energy supplies. In order to have energy for their uncontrolled proliferation, cancer cells reprogramme their glucose metabolism. Glycolysis is elevated normally under anaerobic conditions and is the major source of energy in malignant tumors, even in presence of oxygen (aereobic glycolisis or Warburg effect), where glucose is converted mainly to lactate rather than being involved in the mitochondrial oxidative phosphorylation [51]. Gln, one of the most abundant amino acid in the human body and also in FF [52], is the second primary metabolite to supply cancer cell proliferation. Moreover, Gln is also an important source of energy for in vitro oocyte maturation and the development of embryo [53]. 

Gln metabolism not only assists in ATP production, but it is also essential for biosynthesis of nucleotides, lipid and proteins, in addition to its role as a regulator for redox balance [54]. A recent study by Wang et al., has shown that Gln, synergistically with norepinephrine, promotes estrogen synthesis in FF of IVF patients, and increases the expression of antioxidant genes, as antioxidant gene IDH1, glutathione peroxidase, and Nrf2. Besides, high levels of Gln improve the function of granulose cells in vitro [55]. On the other hand, it cannot be excluded that the decrease of Gln found in patients with BC is the result of a compensatory mechanism to attenuate oxidative stress [56].

It is well known that oxidative and inflammatory stress, events closely related to cancer [57,58], might reduce cellular antioxidant capacity. Nrf2, a ubiquitously expressed transcription factor which regulates the expression of a lot of genes involved in cellular growth, oxidation and detoxifying response, signaling, and cellular cycle [40,41]. In FF of BC patients, we observed a decreased level of Nrf2; the failure to activate the complex downstream antioxidant enzymes, such as NAD(P)H dehydrogenase quinine 1 (NQO1) and cytoprotective protein SOD, might have determined an increase of CXCL10, a 10 kDa proinflammatory chemokine, that is also a modulator of physiological ovarian function [59]. Numerous cytokines and chemokines were detected in FF, the role of which in reproductive physiology seems crucial [60,61]. Cytokines influence the development and maturation of the follicle, ovulation, formation of the corpus luteum, as well as embryo implantation and maintenance of pregnancy. Chemokines are a subset of cytokines responsible for the directed migration of leukocytes during specific conditions; they are produced by immunocompetent cells as well as non-immune cells including normal ovarian cells, granulosa and theca cells. They promote follicular growth processes, steroidogenesis, tissue remodeling during ovulation, luteinisation, and luteolysis [59]. Dysregulation of cytokine profile, also characterized by increased chemokines, is associated with pathological condition such as premature ovarian insufficiency [62] and polycystic ovary syndrome [63]. Moreover, chemokines have a multifaceted role in tumor generating an immunosuppressive tumor microenvironment [64], including breast cancer in which chemokine CXCL10 contributes to the progression of ductal carcinoma in situ to invasive carcinoma of the breast [65,66].

We demonstrated here that CXCL10 is a potentially valuable biomarker for alterations within FF in BC patients. It cannot be excluded that the increase in CXCL10 may be the consequence of a systemic proinflammatory state linked to the presence of tumor which in turn leads to the recruitment of lymphocytes and induces a local response with an increase in chemokines. On the other hand, it cannot be also excluded that there is a Nrf2 dysregulation in the follicular somatic cells which consequently, leads to an increase in chemokines locally. In both cases, considering the deleterious effects that a chronic inflammatory response might have on oocyte quality, it is appropriate to take this into account in a context such as fertility preservation and other efforts must be made to further investigations.

Although ROC curve analysis for Gln, Nrf2 and CXCL10 suggests their potential as breast cancer biomarkers for diagnostics in FF, further studies with large sample size must be conducted to understand the relevance of these species.

## 5. Conclusions

An NMR-based metabolomics approach in combination with the measurement of protein markers of oxidative stress and inflammatory response could give useful information to evaluate the impact of the tumor pathology on the quality of the oocytes that are cryopreserved in awaiting fertilization. However, in this study we could not report oocyte performance because most of the collected oocytes of our BC patients are still in storage, and we cannot yet report information about subsequent embryo and pregnancy outcome. In addition, as a pilot study, this work has its obvious limitation that is the limited sample size; therefore, further studies should then be performed on a larger cohort of patients. 

## Figures and Tables

**Table 1:** Clinical data of the 26 women participating in the study.

	Breast Cancer	Healthy Control
Number of patients	16	10
Age (years)	33.5 (5.0)	36.0 (4.0)
FSH (UI/mL)	6.8 (1.2)	6.9 (2.0)
AMH (ng/mL)	2.8 (1.5)	4.1 (4.4)
AFC	12.2 (7.0)	13.2 (4.0)
Estradiol (pg/mL) *	599.2 (410.4)	1721.7 (1229.0)
Progesterone (ng/mL)	1.9 (1.1)	1.3 (0.9)
BMI (kg/m^2^)	22.3 (2.8)	22.2 (3.4)
Follicles monitored	13.2 (9.0)	11.8 (4.4)
Total oocytes collected	9.3 (7.3)	8.8 (5.9)
MII oocytes	6.3 (6.3)	6.6 (5.2)
Breast cancer stadiation		
T1N0M0	4	
T1N1M0	1	
T1aN1M0	1	
T2N0M0	4	
T2N1M0	1	
T2N2M0	1	
T3N0M0	2	
T3N1M0	2	

Clinical data are reported as mean values with standard deviation in parentheses. Significance (* *p* < 0.05)*;* FSH = Follicle stimulating hormone; AMH = Anti-Mullerian hormone; AFC = Antral follicle count; BMI = body mass index.

## Data Availability

All data are contained in the article and Supplementary Materials.

## Supplementary Materials

The following supporting information can be downloaded at: https://www.mdpi.com/article/10.3390/metabo13030441/s1, Figure S1: Representative ^1^H-NMR spectra of follicular fluids from an healthy control (A) and a breast cancer patient (B). Figure S2: Partial Least Square Discriminant Analysis score plots obtained from the ^1^H-NMR spectral data on FF of the 26 women examined in the study. For this two-component model the R^2^X value was 0.34 and the Q^2^ value was 0.19. Healthy women (n = 10) were compared with BC patients subdivided in two groups: without and with lymph node metastasis. Healthy controls were represented by red triangle. BC patients are shown in black with different symbols: patients with no lymph node metastasis (n = 10), boxes; patients with N1 (n = 5), and N2 lymph node metastasis (n = 1), triangles and dot, respectively; Table S1: Marker metabolites identified by NMR in follicular fluids of healthy (n = 10) and breast cancer women (n = 16). The average integrals of the NMR bin regions ± standard deviations are reported.
