# Supplementary material for: A Pilot Study on Biochemical Profile of Follicular Fluid in Breast Cancer Patients
Source: Metabolites. 2023 Mar 17;13(3):441. doi: 10.3390/metabo13030441 (PMC10054828; doi:10.3390/metabo13030441)
Supplement: Supplementary file 1 [file metabolites-13-00441-s001.zip › metabolites-2254038-Figures S1 and S2.pdf]

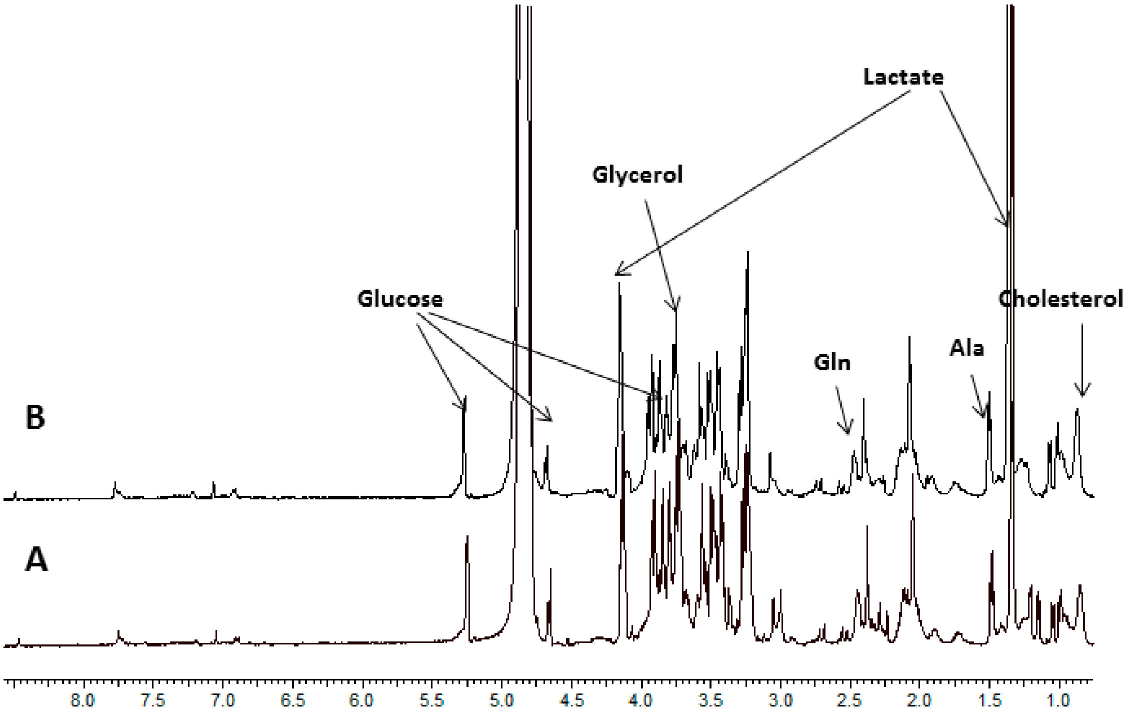

Figure S1. Representative ¹H-NMR spectra of follicular fluids from an healthy control (A) and a breast cancer patient (B).

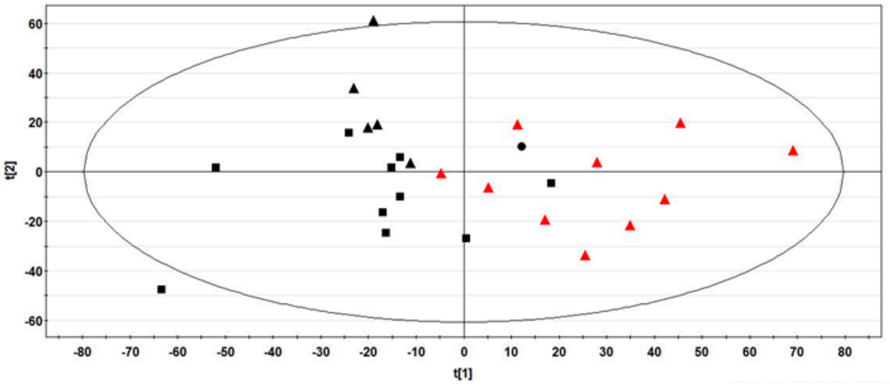

Figure S2. Partial Least Square Discriminant Analysis score plots obtained from the ¹H-NMR spectral data on FF of the 26 women examined in the study. For this two-component model the R^2X value was 0.34 and the Q^2 value was 0.19. Healthy women (n = 10) were compared with BC patients subdivided in two groups: without and with lymph node metastasis. Healthy controls were represented by red triangle. BC patients are shown in black with different symbols: patients with no lymph node metastasis (n = 10), boxes; patients with N1 (n = 5), and N2 lymph node metastasis (n = 1), triangles and dot, respectively.
