# Supplementary material for: A Pilot Study on Biochemical Profile of Follicular Fluid in Breast Cancer Patients
Source: Metabolites. 2023 Mar 17;13(3):441. doi: 10.3390/metabo13030441 (PMC10054828; doi:10.3390/metabo13030441)
Supplement: Supplementary file 1 [file metabolites-13-00441-s001.zip › metabolites-2254038-Table S1.pdf]

Supplementary Table S1. Marker metabolites identified by NMR in follicular fluids of healthy (N = 10) and breast cancer women (N = 16). The average integrals of the NMR bin regions ± standard deviations are reported.

Metabolite	δ (ppm)	Healthy	Oncological	VIP	p-Value
Asparagine	2.88	256.7 ± 259.9	66.9 ± 75.9 ↓	4.0	0.01
Aspartate	3.80	329.9 ± 138.9	237.7 ± 69.7 ↓	2.6	0.03
Cholesterol	0.88	122.1 ± 34.9	87.2 ± 30.5 ↓	1.7	0.01
Choline	3.16	259.6 ± 225.3	101.9 ± 128.6 ↓	3.6	0.03
Creatine	3.92	240.6 ± 61.3	272.8 ± 53.0 ↑	1.5	-
Glutamine	2.48	46.9 ± 13.2	35.2 ± 13.7 ↓	1.0	0.04
Glycerol	3.76	313.8 ± 82.4	354.4 ± 84.9 ↑	1.6	-
-glucose	3.72	283.1 ± 54.5	354.4 ± 84.9 ↑	2.5	0.03
-glucose	3.84	212.8 ± 59.1	268.6 ± 54.6 ↑	2.2	0.02
-glucose	4.64	56.9 ± 26.2	239.4 ± 187.4 ↑	4.1	0.006
Lactate	1.36	643.8 ± 122.7	486.7 ± 188.5 ↓	2.9	-
Lipid	2.76	61.9 ± 71.9	23.2 ± 8.3 ↓	1.7	0.04
Lipid	1.28	165.0 ± 136.8	77.5 ± 22.8 ↓	2.7	0.01
Lysine	1.44	50.6 ± 31.5	26.5 ± 8.3 ↓	1.5	0.007
Proline	2.04	154.2 ± 33.3	178.4 ± 60.3 ↑	1.3	-
TMAO	3.32	48.4 ± 4.5	38.2 ± 9.1 ↓	1.0	0.003

VIP = variable importance in the projection; TMAO = Trimethylamine-N-oxide, - not significant
